# Endovascular treatment of an aortocaval fistula caused by a late type II endoleak

**DOI:** 10.1016/j.jvscit.2024.101436

**Published:** 2024-01-28

**Authors:** Giulio Accarino, Alessandra Benenati, Giancarlo Accarino, Francesco De Vuono, Giovanni Fornino, Gennaro Galasso, Umberto Marcello Bracale

**Affiliations:** a Vascular and Endovascular Surgery Unit, Ospedale San Giovanni di Dio e Ruggi D'Aragona, Salerno, Italy; b Vascular Surgery Unit, Department of Public Health, University Federico II of Naples, Naples, Italy; c Department of Medicine, Surgery and Dentistry, University of Salerno, Baronissi, Italy

**Keywords:** Aortocaval fistula, Endoleak, EVAR

## Abstract

An aortocaval fistula (ACF) is a rare complication of abdominal aortic aneurysms (AAAs) and constitute <1% of all AAAs, which increases from 2% to 6.7% in ruptured AAAs. Unlike other aortic ruptures, most ACFs are not associated with significant blood loss on admission. The traditional treatment strategy has been open surgery, which is associated with a high mortality rate. Endovascular repair has been performed; however, the results are difficult to interpret due to the low incidence of ACFs and the absence of cases reported with a long follow-up duration. We report the case of a 78-year-old man with previous endovascular aneurysm repair performed in 2015, who presented to our emergency department 6 years later with abdominal pain. A computed tomography angiography scan showed type Ia, Ib, and II endoleaks and an ACF. The endoleaks were selectively treated, and the ACF was covered with a polytetrafluoroethylene endograft inserted in the inferior vena cava. In our single-case experience with a medium-term follow-up of 24 months, our treatment was safe and effective for ACF closure, with no further signs of endoleak or graft thrombosis. We conducted a literature review of reported cases in which a covered stent graft was used for ACF treatment. Although no guidelines are currently available regarding this rare late complication after endovascular aneurysm repair, using a covered stent placed in the inferior vena cava to treat an ACF could be a viable option in selected cases.

Aortocaval fistula (ACF) is a rare complication of abdominal aortic aneurysms (AAAs) and constitute <1% of all AAAs, increasing from 2% to 6.7% in ruptured AAAs.^1^^,^^2^ Penetrating traumatic injury and recent lumbosacral laminectomy are otherwise the most common causes of abdominal arteriovenous fistulas.^3^ ACF was first described by Symes in 1831, and Dr. Cooley reported the first successful repair in 1955.^4^ Most ACFs present without a massive hematoma because the aortic rupture shunts into the inferior vena cava (IVC).^5^ The most common symptoms are determined by rapid arteriovenous shunting, which causes secondary venous hypertension, and include abdominal bruit, oliguria, tachycardia, hypotension, and right heart failure.^2^^,^^6^ Early treatment can improve survival from 25% to 50%.^7^ Computed tomography angiography (CTA) is the primary diagnostic modality. Invasive methods, including arteriography and venography with pressure gradient assessment, can also be used.^8^^,^^9^ Failure to demonstrate the ACF by CTA can occur because of the timing of the scans or the small size of the ACF.^4^ The traditional method of repair has been open surgery, which is associated with a high mortality rate.^1^ Endovascular repair has been performed; however, results are difficult to interpret due to the low incidence of ACFs and the absence of cases reported with a long follow-up. We present the case of a patient who underwent an endovascular repair of an ACF using an endograft implanted in the IVC. The patient provided written informed consent for the report of his anonymized case details and imaging studies.

## Case report

**Fig 1:**
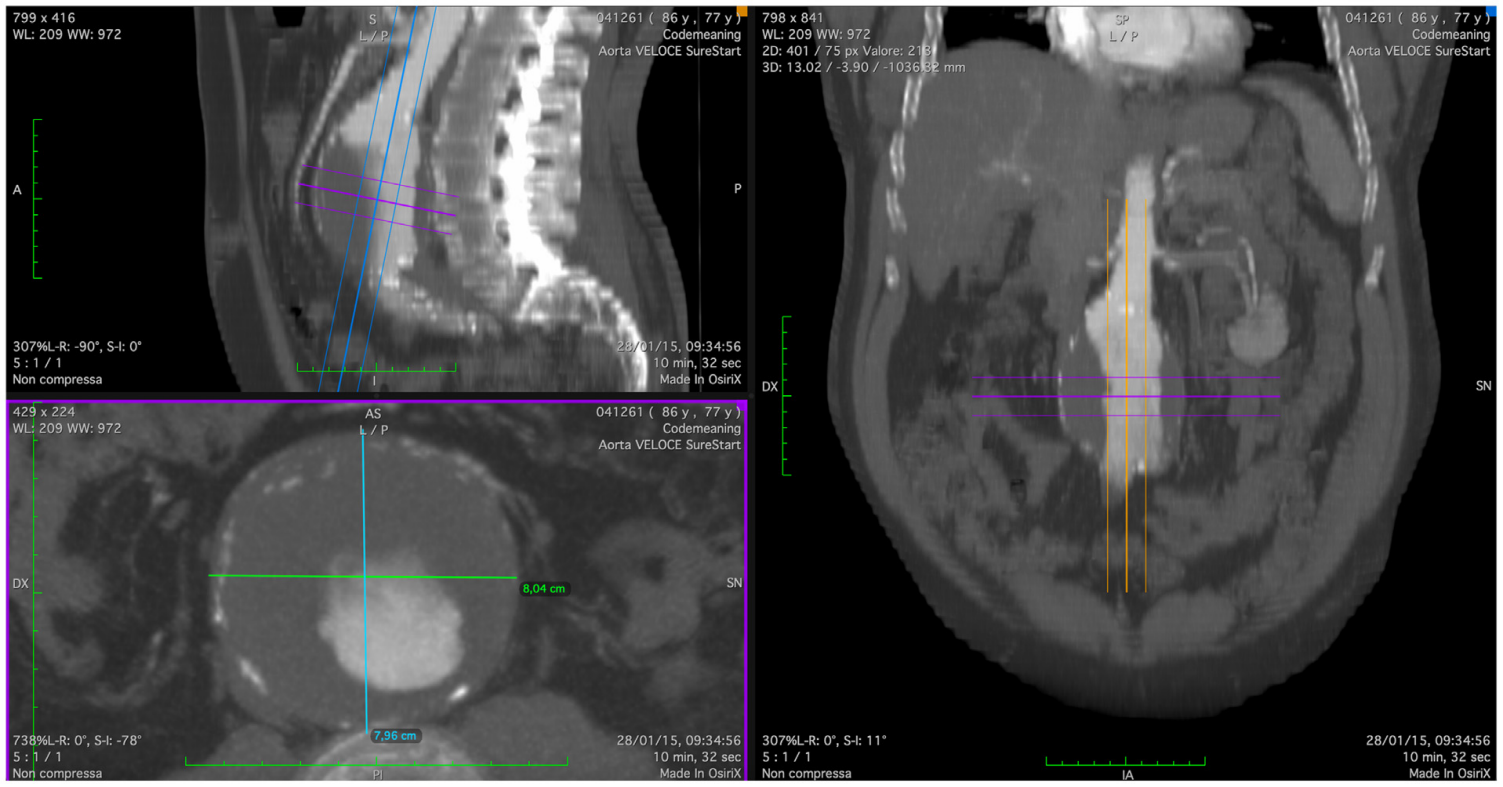
Multiplanar reconstruction of the abdominal aortic aneurysm (AAA) in 2015.

**Fig 2:**
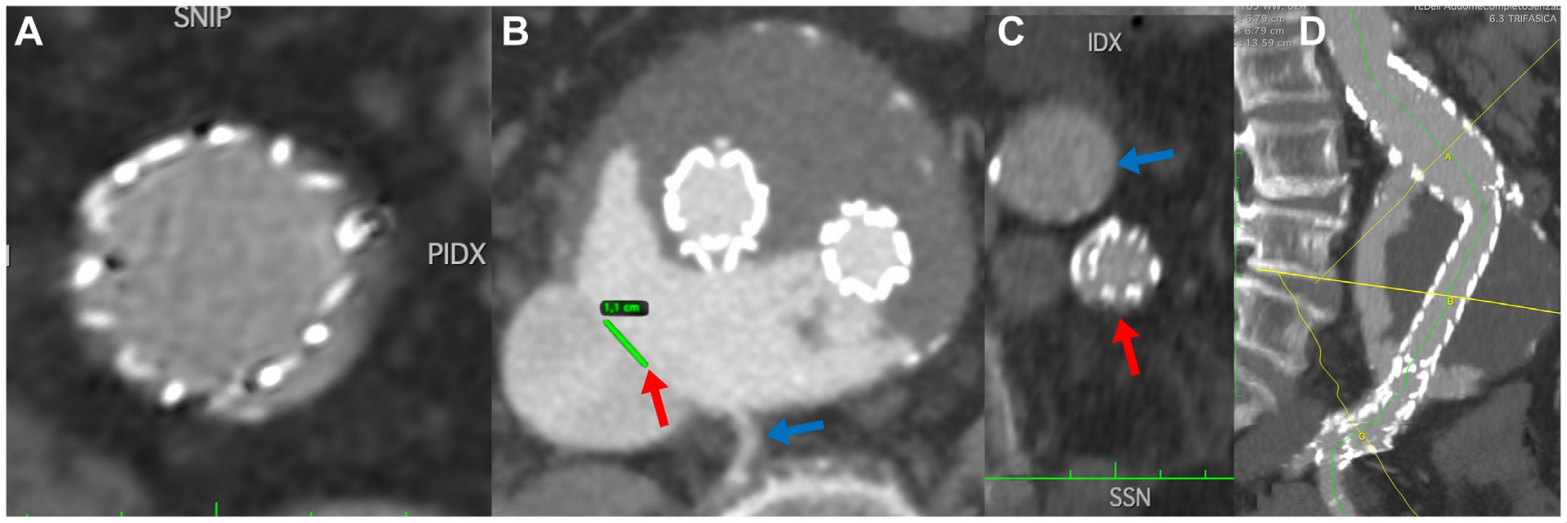
Three endoleaks (**A,** Ia; **B,** II; and **C,** Ib) and an aortocaval fistula (ACF; **D**).

**Fig 3:**
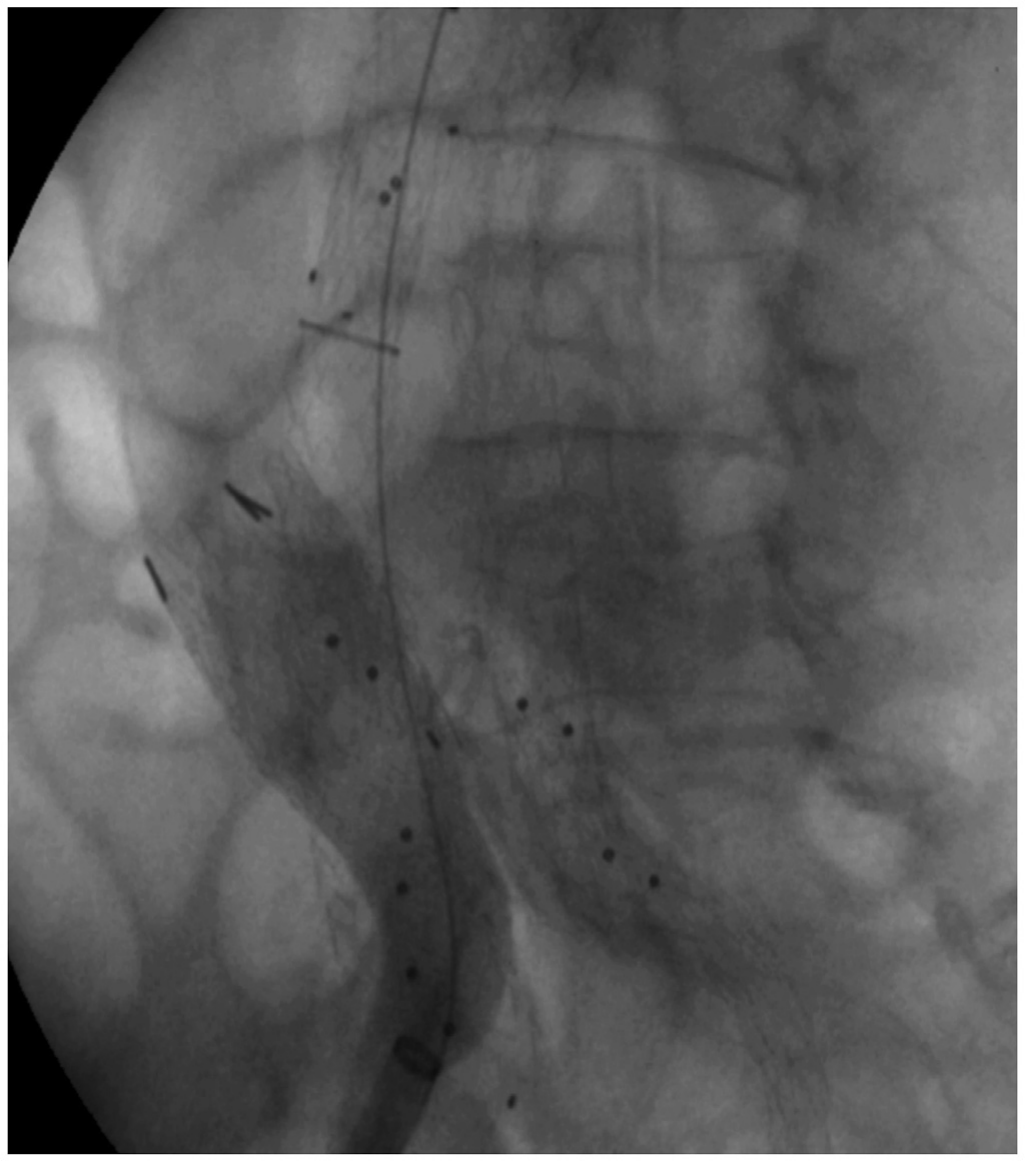
Angiogram showing the endograft in the inferior vena cava (IVC) and aortocaval fistula (ACF) sealing.

**Fig 4:**
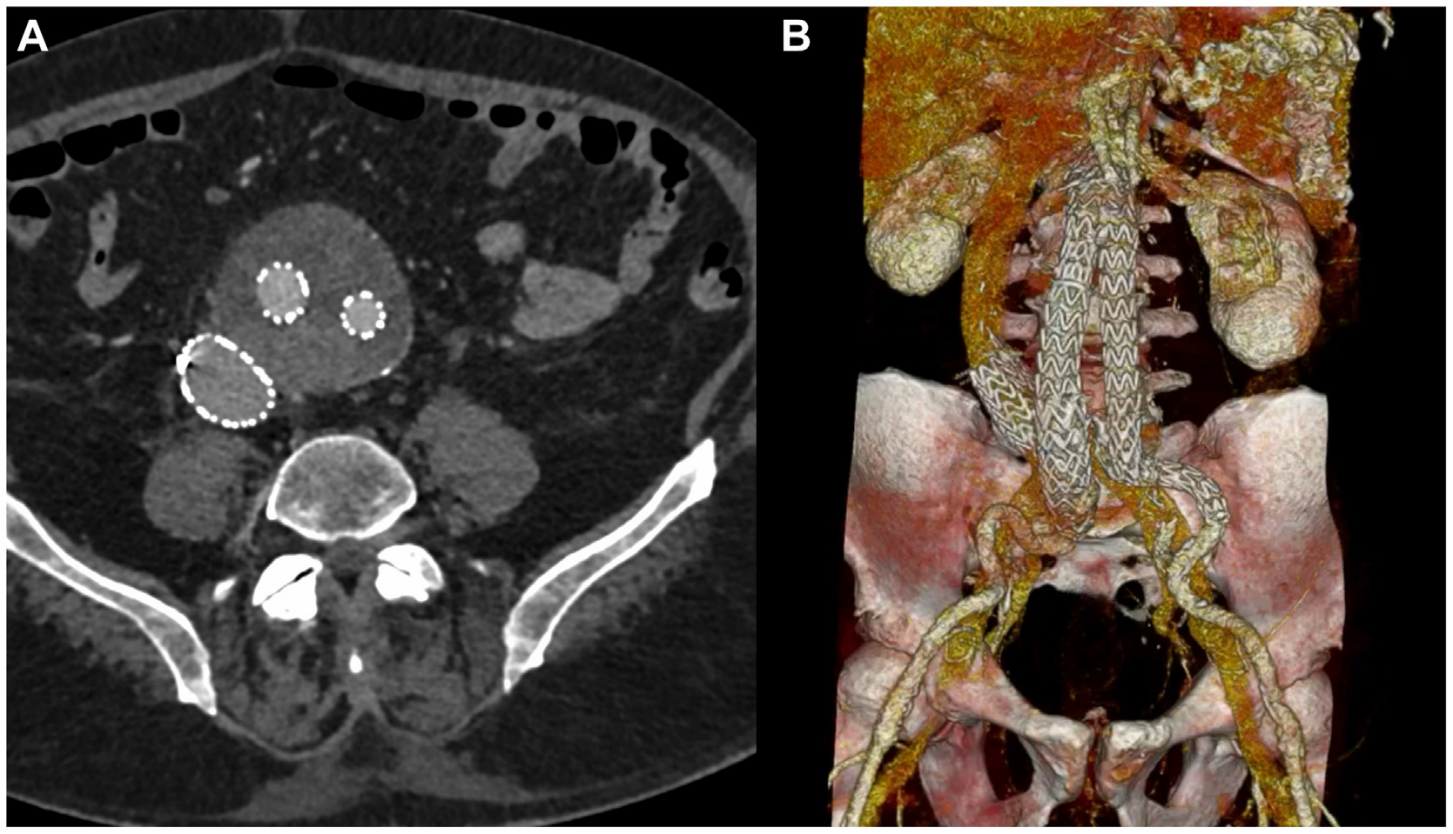
Follow-up computed tomography angiography (CTA) at 24 months postoperatively showing no signs of endoleak or aortocaval fistula (ACF; **A**) and perfect patency of all endografts implanted (**B**).

A 78-year-old man presented to our attention with an 80-mm AAA (Fig 1). His medical history included arterial hypertension, dyslipidemia, and chronic obstructive pulmonary disease. The patient had no previous abdominal surgery or deep vein endovascular treatment. CTA showed an AAA with a tapered 27-mm and 14-mm long neck, a patent inferior mesenteric artery, and four patent lumbar arteries. The blood test results showed dyslipidemia. In March 2015, he underwent endovascular aneurysm repair (EVAR). The devices used were a main body 28 mm proximal, 16 mm distal and 166 mm long on the ipsilateral side and 16 and 20 mm proximal and distal diameter, 124 mm long bell bottom stent grafts (Medtronic Vascular). The distal sealing zone on the right side was the proximal common iliac artery and on the left side was the distal portion of the common iliac artery. We found an intraoperative type IIb endoleak between the inferior mesenteric artery and a lumbar artery that we chose to monitor during follow-up. The hospitalization stay was 4 days, and the patient was discharged on the second postoperative day. The patient was lost to follow-up for CTA scans and clinical examinations between the procedure and 2021, when he presented to our emergency department with abdominal pain. On that day, a CTA was performed and showed three endoleaks (types Ia, IIa, and Ib on the left side; Fig 2, *A-C*) and an ACF (Fig 2, *D*). An echocardiogram showed that the IVC was not collapsing with breathing, and the systolic pulmonary artery pressure was 70 mm Hg. Given the patient's preference for noninvasive treatment and the substantial worsening of chronic obstructive pulmonary disease, endovascular strategies were the only options. In July 2021, the patient underwent a second procedure to treat the endoleaks and repair the ACF. Under locoregional anesthesia to reduce the surgical risk, we exposed both femoral arteries. We deployed a 32- by 49-mm Endurant proximal aortic extension (Medtronic Vascular), which was fixed with four HeliFX endoanchors (Medtronic Vascular) to treat the type Ia endoleak. Subsequently, the endograft was extended distally on the right side with an Endurant 20-20-82 stent graft landing in the distal common iliac artery and Endurant 16-10-93 stent graft (Medtronic Vascular) on the left side with a distal landing zone in the external iliac artery to seal the severely stenotic origin of the internal iliac artery, with no further procedures. A right common femoral vein puncture was performed, and a 18F DrySeal sheath (W. L. Gore & Associates, Inc) was advanced into the IVC. After identifying the exact point of the large ACF, we deployed a 32- by 45-mm aortic extension (W. L. Gore & Associates, Inc) that covered the IVC lesion without issue (Fig 3). At the final angiogram, no signs of an endoleak or ACF were found, and there was normal flow in the IVC. Postoperatively, the patient developed acute lung failure that required 7 days of hospital stay. To support the patency of the endograft implanted in the IVC, lifelong treatment with rivaroxaban 2.5 mg was prescribed twice daily. His subsequent postoperative course was unremarkable. At the last available follow-up examination (July 2023), the patient had no symptoms, and the follow-up CTA showed no signs of endoleak or ACF and perfect patency of all the endografts (Fig 4).

## Discussion

An ACF that appears later in the follow-up of an EVAR patient is exceedingly rare and has been reported in only a few cases to date.^4^, ^5^, ^6^, ^7^, ^8^ Our patient presented to our emergency department with abdominal pain, which seemed unrelated to the ACF itself. However, because we noted three different endoleaks that were providing flow to the ACF in the absence of signs of endograft infection, we deemed urgent treatment was necessary. We measured the IVC tear at 11 mm (Fig 2, *B*). At the time of treatment, we were unable to identify a specific threshold for ACF treatment. Because the traditional open method for treating this condition was not a viable choice due to both the patient's preference and his precarious respiratory condition, we were left only with endovascular strategies.^9^ Although the ACF might have become sealed on its own, we chose not to stage the procedure by treating the type I endoleaks first. We would still have had to treat the type II endoleak that could provide flow to the ACF. We believed that performing a single-stage procedure would reduce the surgical risk for such a frail patient.

Our patient presented with three endoleaks. Nevertheless, the aneurysmal sac expansion was negligible (<5 mm) and was probably due to the release of pressure caused by the endoleaks in the aneurysmal sac to the caval circulation. Although extensively coiling the aneurysmal sac could have been a solution, as reported for some cases,^10^^,^^11^ considering the extension of the ACF, we chose to discard this solution owing to the risk of spiral migration through the ACF. After discussing the use of a vascular plug, we chose a covered stent graft because of the force the vascular plug could have exerted on the injured IVC wall.

A review reported in 2021 by Smeets et al^12^ identified 35 patients in whom a covered stent was used to treat bleeding arising from the IVC or iliac veins. In 14 patients, grafts originally intended for the abdominal aorta were used, and in 4 patients, an endograft for thoracic aneurysm repair was used. When choosing the device, both the tissue of the endoprosthesis and the type of structure must be considered because these factors influence the outcome. It is crucial to consider that the blood flow in the IVC is inconstant and at low velocity compared with arterial flow. Moreover, the structure of the ICV is not rigid and often tends to collapse, greatly increasing the risk of thrombosis. Commercially available aortic endografts are covered in polytetrafluoroethylene or polyester. Between the two, polytetrafluoroethylene seems to be less prone to adverse events when used as a bypass graft to treat peripheral artery disease or for EVAR,^13^^,^^14^ and these findings guided our stent graft choice.

Matteo et al^15^ proposed a mathematical method for stent diameter sizing in the IVC, and Melas et al^16^ applied 20% oversizing, which was confirmed by a systematic review reported in 2021.^12^ In all reported cases, no complications developed related to the endograft diameter; thus, it is not possible to give any recommendations to date. We chose to apply 30% oversizing based on the CTA findings to ensure enough radial force to seal the lesion and avoid migration. Although the endograft length does not seem to influence thrombus formation,^12^ to reduce venous coverage, we selected the shortest 45-mm abdominal aortic tube, despite the presence of radial hooks that could injure the IVC wall.

Our patient was discharged with relatively long aortic and short IVC coverage. In the context of venous coverage with an endograft, no guidelines are currently available. However, a Delphi consensus published in 2018 about antithrombotic therapy after venous stenting to treat a venous occlusion concluded that anticoagulation therapy should be preferred to antiplatelet therapy.^17^ To date, our guidelines have no recommendations regarding postoperative pharmacologic treatment, leaving this choice to the physician.^12^ To reduce the risk of graft thrombosis, we prescribed low-dose rivaroxaban twice daily.^12^

**Fig 5:**
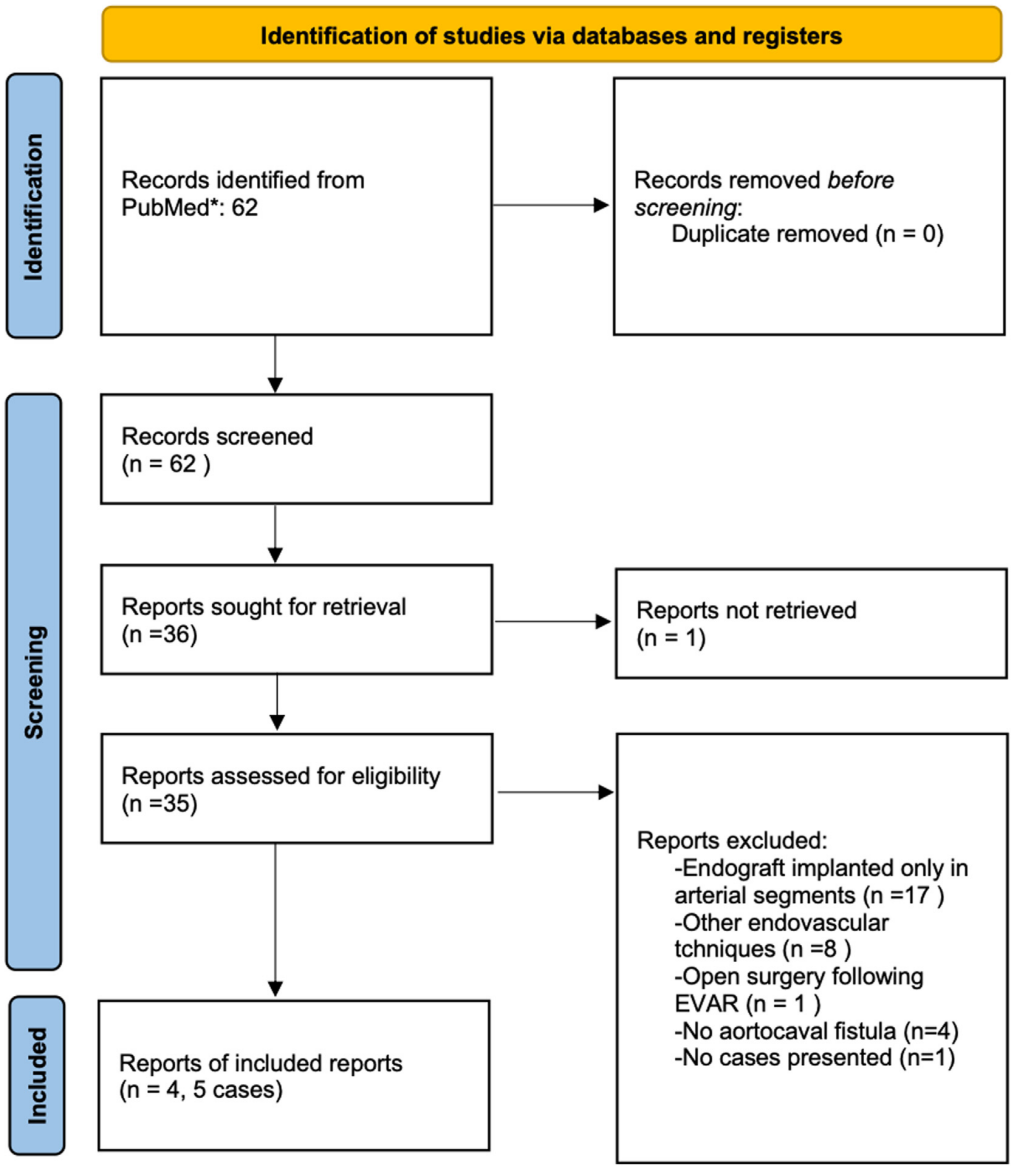
PRISMA (preferred reporting items for systematic reviews and meta-analyses) 2020 flowchart for literature review of cases that reported an endograft implanted in the inferior vena cava (IVC) to treat an aortocaval fistula (ACF).

We conducted a systematic review of all cases reported in PubMed that reported an endograft implanted in the IVC to treat an ACF (Fig 5). We found four reports and five cases^16^^,^^18^, ^19^, ^20^ (Table).

**Table:** Cases reporting stent graft use to treat an aortocaval fistula (ACF) after previous or concomitant endovascular aneurysm repair (EVAR)

Variable	Pt. No.				
	1	2	3	4	5
Age, years	76	65	73	84	73
Previous/concomitant EVAR	Previous	Concomitant	Concomitant	Concomitant	Concomitant
Signs of heart failure	Yes, mild	Yes, shock	Yes, severe	No	No
ACF dimension	NA	NA	NA	NA	NA
Endograft in IVC	Lemaitre UniFit	Gore TAG	Gore cTAG, Endologix PowerLink	Gore cTAG	Gore cTAG, Endologix PowerLink
Endograft dimensions, mm	24 × 100	NA	NA	NA	NA
Outcome	Good	Early compression; good after endograft ballooning	Good	Good	Good
Follow-up, months	36	3	None	None	None
Prescribed medications	Acenocumarol and clopidogrel	NA	NA	NA	NA

*IVC*, inferior vena cava; *NA*, not available; *Pt. No.*, patient number.

## Conclusions

In our single-case experience with medium-term follow-up (24 months), our treatment was safe and effective for ACF closure. Although no guidelines are currently available regarding this rare complication after EVAR, using an IVC-implanted covered stent to treat an ACF could be a viable option in selected cases. Further research with longer follow-up and pharmacologic therapy standardization are needed to establish new treatment strategies for such delicate clinical settings.

## Disclosures

None.

## References

[1] Warning K., Houlind K., Ravn H. (2016). Aortocaval fistula (ACF) in patients operated for ruptured acute aorta aneurysm (rAAA): a surgical challenge. Eur J Vasc Endovasc Surg.

[2] Schmidt R., Bruns C., Walter M., Erasmi H. (1994). Aorto-caval fistula--an uncommon complication of infrarenal aortic aneurysms. Thorac Cardiovasc Surg.

[3] Ribeiro T., Ferreira R.S., Catarino J. (2021). Primary aortocaval fistula in ruptured abdominal aortic aneurysm — institutional experience and literature review. Eur J Vasc Endovasc Surg.

[4] Mouawad N.J., Quarrie R., Starr J. (2020). Acute aortocaval fistula secondary to chronic type 1 B abdominal aortic aneurysm endoleak. Int J Angiol.

[5] Khan A., Vasudevan T. (2017). Bridging stent repair of type III endoleak causing aortocaval fistula after branched aortic endovascular repair. J Vasc Surg Cases Innov Tech.

[6] Hetzel G., Gabriel P., Rompel O., Ritter W., Raithel D. (2006). Aortocaval fistula after stent-graft repair. J Endovasc Ther.

[7] Calligaro K.D., Savarese R.P., DeLaurentis D.A. (1990). Unusual aspects of aortovenous fistulas associated with ruptured abdominal aortic aneurysms. J Vasc Surg.

[8] Rajendran V., Sundararajan K., Sawka A. (2018). Aortocaval fistula presenting as type 2 acute myocardial infarction in a patient with severe arteriopathy. Indian J Crit Care Med.

[9] Dakis K., Nana P., Kouvelos G. (2023). Treatment of aortocaval fistula secondary to abdominal aortic aneurysm: a systematic review. Ann Vasc Surg.

[10] De Boodt H., Pardon H.E., Gellens P., Maleux G., Marrannes J. (2020). Balloon-assisted transcaval embolization of a type II endoleak associated with an aortocaval fistula after endovascular aortic repair. J Vasc Surg Cases Innov Tech.

[11] Ascoli Marchetti A., Oddi F.M., Diotallevi N., Battistini M., Ippoliti A. (2020). An unusual complication after endovascular aneurysm repair for giant abdominal aortic aneurysm with aortocaval fistula: high bilirubin levels. SAGE Open Med Case Rep.

[12] Smeets R.R., Demir D., van Laanen J., Schurink G.W.H., Mees B.M.E. (2021). Use of covered stent grafts as treatment of traumatic venous injury to the inferior vena cava and iliac veins: a systematic review. J Vasc Surg Venous Lymphat Disord.

[13] Russu E., Mureșan A.V., Ivănescu A.D. (2023). Polytetrafluorethylene (PTFE) vs. Polyester (Dacron ®) grafts in critical limb ischemia salvage. Int J Environ Res Public Health.

[14] Labarrere C.A., Dabiri A.E., Kassab G.S. (2020). Thrombogenic and inflammatory reactions to biomaterials in medical devices. Front Bioeng Biotechnol.

[15] Matteo J., Hood P., Hulsberg P.C. (2018). Larger sizes matter more! Applying the matteo mathematics method for endovascular aortic bifurcation reconstruction to large venous vascular repair. Cureus.

[16] Melas N., Saratzis A., Saratzis N., Lazaridis I., Kiskinis D. (2011). Inferior vena cava stent-graft placement to treat endoleak associated with an aortocaval fistula. J Endovasc Ther.

[17] Milinis K., Thapar A., Shalhoub J., Davies A.H. (2018). Antithrombotic therapy following venous stenting: international Delphi Consensus. Eur J Vasc Endovasc Surg.

[18] Siada S.S., Malgor E.A., Malgor R.D., Colvard B.D., Jacobs D.L. (2020). Percutaneous endovascular repair of a ruptured extent III thoracoabdominal aortic aneurysm with bilateral large common iliac aneurysms and aortocaval fistula. Ann Vasc Surg.

[19] Sultan S., Zaki M., Alawy M., Elkassaby M. (2014). Aortic and inferior vena cava bifurcated stent graft application in the endovascular management of a ruptured abdominal aortic aneurysm with an aortocaval fistula. J Vasc Surg.

[20] Elkassaby M., Alawy M., Zaki M., Hynes N., Tawfick W., Sultan S. (2014). Total endovascular management of ruptured aortocaval fistula: technical challenges and case report. Vascular.

